# Perylene Dye as a Measure of Dynamic Free Volume, Fragility, and Dynamic Glass Transition in Thin Polystyrene Films

**DOI:** 10.1021/acs.macromol.5c03244

**Published:** 2026-02-26

**Authors:** Sarita Yadav, Connie B Roth

**Affiliations:** Department of Physics, 1371 Emory University, Atlanta, Georgia 30322, United States

## Abstract

We demonstrate the ability of perylene dye to capture the high frequency GHz vibrational density fluctuations of polymer matrices in thin polystyrene (PS) films with decreasing film thickness. From perylene’s temperature dependent fluorescence characteristics that are observed to be proportional to the polymer’s relaxation time τ­(*T*), a perylene determined glass transition temperature $T_{g}^{\mathrm{peryl}}$ is defined by the change in temperature dependence on cooling from a non-Arrhenius to Arrhenius behavior at the polymer’s *T*_g_. We find $T_{g}^{\mathrm{peryl}}$ behaves like a dynamic measure of *T*_g_ that is independent of film thickness *h* down to 15 nm. Fits to a Williams–Landel–Ferry (WLF) functional form above *T*_g_ quantify a decrease in fragility with decreasing film thickness and demonstrate that the relaxation dynamics are significantly faster in thin films, with the average relaxation time at *T*_g_ decreasing by many orders of magnitude. These results are contrasted with those from pyrene dye that exhibit *T*_g_(*h*) decreases consistent with thermodynamic measures of *T*_g_ like ellipsometry.

## Graphical abstract

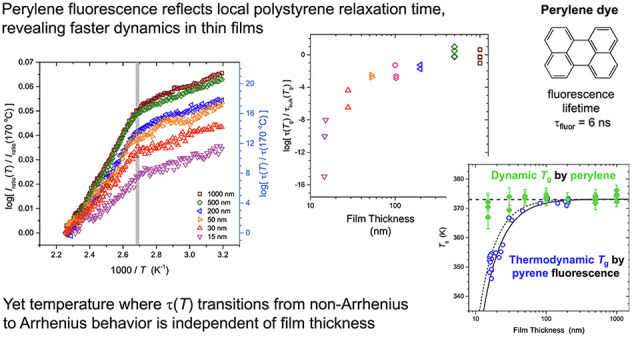

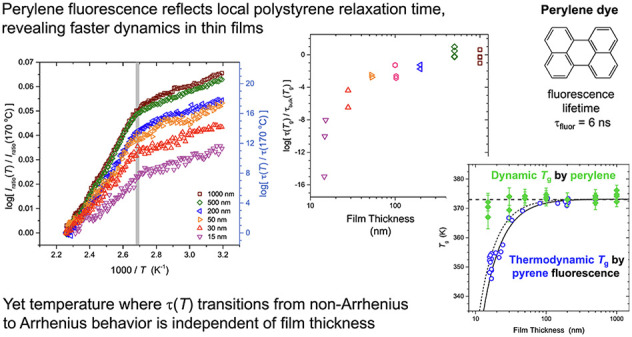

## 1 Introduction

Numerous industrial applications involve polymer thin films spanning fields such as nanolithography, batteries, and filtration membranes, where understanding of the material’s behavior is necessary to the application’s design.^1–4^ As the thickness of polymer films decrease, changes in various different material properties have been shown to arise, such as for the glass transition temperature,^5–7^ α-relaxation time,^8,9^ fragility,^8,10–12^ physical aging,^13–15^ refractive index,^16,17^ modulus,^18–22^ viscosity,^23,24^ and mean square displacement.^25–30^ These material property changes have been associated with dynamical perturbations caused by the presence of interfaces like the free surface.^1,31–36^ An important open question at present is the degree to which different material properties maintain the same relative interrelation as in the bulk when in a thin film that is perturbed by interfacial effects?

In bulk systems, different experimental measures of the glass transition are known to be correlated. The temperature at which a thermodynamic state variable such as volume or density changes on cooling from the equilibrium liquid state to a nonequilibrium glass is generally the same as the temperature at which the super-Arrhenius slowing down of the cooperative segmental relaxation time τ­(*T*) on cooling reaches 100 s. In contrast, for thin films and other nanoconfined systems, it has been experimentally shown that such different measures of the glass transition temperature *T*_g_ exhibit different film thickness dependencies with decreasing thickness.^7,37–39^ Thermodynamic measures of *T*_g_, such as the temperature at which a film’s thermal expansion changes exhibit large decreases in *T*_g_(*h*) with decreasing film thickness *h*,^5^ while dynamic measures of *T*_g_, such as the position of τ­(*T*) measured by dielectric spectroscopy, do not show comparable changes in thin films.^7^ Several possible explanations have been proposed for this inconsistency.^37,38,40,41^ One possible explanation suggested by a computational analysis of local depth-dependent dynamics in thin films obtained using molecular dynamics simulations is that how local properties are averaged across the entire film, resulting in the different average measures of *T*_g_(*h*), may be weighted differently for thermodynamic versus dynamic quantities.^40^ Given these unresolved observations, there is an important need to develop new experimental techniques that can be adapted to local depth-dependent measurements in order to provide a more complete map of dynamical gradients in thin films. Toward this end, here, we investigate the ability of perylene dye to capture changes in the dynamical properties of polystyrene (PS) thin films with decreasing film thickness.

Pyrene dye is a well-established fluorescent probe of the glass transition. The *T*_g_ values obtained by pyrene’s temperature dependent changes in fluorescence intensity have been shown to agree with thermodynamic measures of the glass transition such as differential scanning calorimetry (DSC) in bulk systems and ellipsometry in thin films that quantifies the film’s temperature dependent volume changes.^31,42–44^ The changes in *T*_g_(*h*) with decreasing film thickness obtained by pyrene fluorescence have been shown to agree well with those obtained by ellipsometry exhibiting the same functional form, where *T*_g_(*h*) is observed to decrease by ≈30 K for 15 nm thick PS films.^31,43^ When covalently attached to a high molecular weight polymer, the pyrene dye can be restricted to a thin probe layer creating a local measure of this thermodynamic *T*_g_ as the fluorescence signal is arising only from the fluorescently labeled layer within a given multilayer assembly.^31^ By positioning the pyrene-labeled layer at different depths *z* from an interface, a complete map of depth-dependent *T*_g_(*z*) changes in the glass transition can be constructed.^45–49^ Over the past two decades, pyrene dye as a local probe has yielded significant insights into local *T*_g_(*z*) gradients generated by perturbations from different types of interfaces.^31,35,45–47,49–62^ For example, local pyrene fluorescence measurements were the first to provide direct evidence that the free surface is the primary source of *T*_g_(*h*) reductions in thin PS films.^31,35^ Overall, fluorescence measurements have a huge advantage over the other experimental techniques as signal results exclusively from where the dye is placed in the sample, and only trace levels of dye are needed to obtain good signal strength.

Perylene, the fluorescent dye that we use in this study, is a polycyclic aromatic hydrocarbon with similar chemical structure and size to pyrene, but it has notably different fluorescence characteristics. Perylene is known for its high quantum yield, short excited state lifetime, and good thermal stability at high temperatures,^63^ making it a promising candidate for local fluorescence measurements. Molecular dyes that are derived from perylene are extensively used in a host of applications from organic light-emitting diode displays to biological research.^64–68^ In polymers, perylene has been employed as a molecular thermometer,^69–71^ to create temperature-sensitive paints,^72,73^ and perylene-based dyes have also been used as rotational probes to measure segmental dynamics.^74–86^ Recently, Han and Roth demonstrated perylene’s fluorescence sensitivity to investigate polymer dynamics by doping it in four different polymer matrices: polystyrene (PS), poly­(methyl methacrylate) (PMMA), poly­(2-vinylpyridine) (P2VP), and polycarbonate (PC).^87^ From an examination of changes in the fluorescence emission spectrum with temperature, they found that the temperature dependence of the intensity ratio of the first peak relative to a temperature independent region dubbed the self-referencing region (SRR) followed the relaxation behavior of the respective polymer in all four different polymer matrices, specifically transitioning at the *T*_g_ of the polymer matrix (i.e., varying from 99 °C for P2VP to 152 °C for PC). In the liquid state above the polymer’s *T*_g_, a non-Arrhenius temperature dependence of the fluorescence intensity was observed that agreed well with the Williams–Landel–Ferry (WLF) equation, whereas an Arrhenius temperature dependence was observed in the glassy state below the polymer’s *T*_g_.^87^ This observed temperature dependence is consistent with that of the polymer’s segmental relaxation time τ­(*T*), strongly indicating that perylene dye is sensitive to the local surrounding polymer dynamics and is effectively captured by its fluorescence emission intensity. This gives us compelling evidence that perylene dye could be effectively used for measuring the local dynamic properties of the polymer matrix.

In this work, we investigate the ability of perylene fluorescence spectroscopy to capture the dynamic material property changes of supported polystyrene (PS) thin films due to the presence of the free polymer–air interface. Specifically, we map how the temperature dependence of perylene fluorescence changes as the thickness of the PS films is decreased, thereby increasing the dynamical perturbation from the free surface on the average film properties. Supported thin films of PS are a system that has been extensively studied in the literature, making it an excellent system for which to benchmark our understanding of how perylene fluorescence characterizes polymer properties in nanoconfined systems. The changes in fluorescence intensity with temperature are quantified through an intensity ratio of the first peak that has the highest intensity to a part of the emission spectrum that is independent of temperature, accounting for any changes in intensity due to photobleaching. The temperature dependence of the fluorescence intensity is fit to a WLF functional form in the polymer’s liquid state and an Arrhenius temperature dependence in the glassy state, allowing us to define a perylene determined glass transition temperature $T_{g}^{\mathrm{peryl}}$ at the intersection of where this temperature dependence changes. We show how perylene’s temperature dependence can be correlated with the τ­(*T*) dependence of PS, which we believe arises because of perylene’s short nanosecond fluorescence lifetime making the dye sensitive to the polymer’s molecular density fluctuations at GHz frequencies. As such, perylene appears to provide an effective measure of the polymer’s dynamic free volume, where we believe $T_{g}^{\mathrm{peryl}}$ may be associated with a dynamic measure of the glass transition $T_{g}^{\mathrm{dyn}}$ . The non-Arrhenius WLF behavior in the liquid state is observed to weaken with decreasing PS film thickness indicating faster dynamics and a reduction in the fragility of the thin films, in agreement with existing literature studies.^8,10–12^ Interestingly, we find $T_{g}^{\mathrm{peryl}}$ = $T_{g}^{\mathrm{dyn}}$ to be independent of PS film thickness down to 15 nm, in contrast to pyrene’s thermodynamic-like glass transition $T_{g}^{\mathrm{pyr}}$ that decreases with decreasing film thickness in PS following literature *T*_g_(*h*) values.^42,43,88^ This indicates that future measurements contrasting perylene’s response with pyrene’s may be able to provide insight into the differences of how dynamic and thermodynamic measures of *T*_g_ differ in nanoconfined systems.^7,37–40^

## 2 Experimental Methods

Polystyrene (PS) with a molecular weight *M*_w_ = 650 kg/mol and *M*_w_/*M*_n_ = 1.06 was used as received from Pressure Chemical. Perylene-doped PS solutions were prepared by dissolving PS in toluene with trace levels of perylene dye (0.2 wt % relative to PS mass). In the dried polymer film sample, this dye concentration is equivalent to 0.081 mol % relative to the PS repeat unit. The optical density of the samples was always less than 0.032 even for the thickest 1000 nm films, well below the absorbance criteria of <0.05 to avoid fluorescence reabsorption.^89^ We also verified that the perylene fluorescence emission spectrum was independent of dye concentration between 0.1 and 1.0 wt % (0.04 to 0.4 mol %), ensuring that we are in the dilute regime. No dye aggregation in the form of excimer fluorescence was observed from perylene at these low dye concentrations, indicating the perylene dyes were isolated and well dispersed within the polymer matrix. Polymer films were made by spin-coating the solution onto fused silica substrates for fluorescence measurements. The spin speed needed to obtain the desired thickness was determined by spin-coating films onto silicon wafers for ellipsometry measurements using the same solution. Spectroscopic ellipsometry (Woollam M-2000) was used to obtain the film thickness by measuring changes in the light polarization, where the ellipsometric angles Ψ­(λ) and Δ­(λ) were fit to an optical layer model consisting of air, the polymer film of thickness *h*, and a semi-infinite silicon substrate with 1.25 nm thick native silicon-oxide layer.^90^ The refractive index of the polymer film over the wavelength range λ = 400–1000 nm was modeled using a Cauchy layer model with $n\left(\lambda \right)=A+\frac{B}{\lambda^{2}}$ .^16^ The thicknesses of the films investigated in this study are 998 ± 10, 505 ± 10, 198 ± 2, 102 ± 5, 53 ± 1, 28 ± 1, and 14.8 ± 0.3 nm.

Steady-state fluorescence measurements were taken with a Photon Technology International QuantaMaster spectrometer. During the measurement, the polymer films supported on the fused silica substrates were covered by a clear quartz piece to limit photobleaching of the dye by oxygen. The perylene dyes doped in the PS films were excited at 394 nm using a xenon arc lamp with excitation bandpasses of 3 nm and emission bandpasses of 5 nm.^87^ Prior to measurement, the samples were initially annealed at 130 °C for 20 min within the fluorometer heater (Instec HCS402) to remove the thermal history of the sample. Then, the samples were ramped to 170 °C and stabilized to initiate the measurement by collecting fluorescence intensity on cooling at 1 °C/min from 170 to 40 °C. The emission wavelengths collected during these temperature runs were 444, 445, and 446 nm for the first peak and 438, 439, and 440 nm for the temperature-invariant self-referencing region (SRR), where intensity was sampled for 3 s at each wavelength corresponding to a total of 18 s of illumination during every 60 s period of data collection. At the end of the cooling run, all of the samples were reheated to the same initial temperature of 130 °C, where a full emission spectrum was collected again to compare with a spectrum collected at the beginning of the run, verifying that negligible photobleaching had occurred.

A few fluorescence measurements were also taken using pyrene dye doped into PS films, also at 0.2 wt %.^42^ For these measurements, pyrene was excited at 332 nm with 3 nm bandpasses, where the full emission spectrum from 350 to 450 nm using 5 nm bandpasses was collected at 0.3 s/nm every 5 °C on cooling the sample at 1 °C/min from 130 to 50 °C. As before, the sample was initially held at 130 °C for 20 min to remove thermal history, and the sample was reheated to 130 °C at the end of the run to ensure photobleaching was negligible. The integrated fluorescence intensity under the full emission spectrum was plotted as a function of temperature, where the intersection of linear fits to the data in the liquid and glassy regimes were used to identify a pyrene determined glass transition temperature $T_{g}^{\mathrm{pyr}}$ .^31,42,88^ From an average of two samples on films of bulk thickness (≈500 nm), the bulk glass transition temperature of PS was determined to be $T_{g}^{\mathrm{bulk}}$ = 373 ± 1 K.

## 3 Results and Discussion

### 3.1 Quantifying the Temperature Dependence of Perylene’s Fluorescence Emission Spectrum

We start in Figure 1a by showing the full fluorescence emission spectrum as a function of wavelength (425–525 nm) for perylene doped in a 1000 nm thick PS film supported on silica. Spectra collected at three different temperatures of 170, 130, and 40 °C, are graphed demonstrating the increase in the peak intensity and decrease in the trough intensity observed with decreasing temperature. Nonradiative energy loss of the perylene dye resulting from thermal agitation of the surrounding polymer (such as intermolecular and intramolecular vibrations, rotations, and density fluctuations) increases with increasing temperature resulting in a decrease in fluorescence intensity at higher temperatures.^63^ No lateral shift in the fluorescence spectrum is observed with varying temperature. As such, there are locations of the spectrum that are temperature invariant as described by Han and Roth,^87^ where the first temperature-invariant region to the left of the first peak was dubbed the self-referencing region (SRR). Following this previous work,^87^ we define an intensity ratio *I*_ratio_ = *I*_peak_/*I*_SRR_ based on the ratio of the first peak intensity to the intensity at the SRR. In practice, *I*_ratio_ is calculated by summing the intensities of the first peak between 444–446 nm and the SRR region between 438–440 nm, whose wavelengths are highlighted in Figure 1. The benefit of dividing by *I*_SRR_ is that *I*_ratio_ is independent of changes in the fluorescence intensity that might be caused by photobleaching or instrument variability. In this way, *I*_ratio_ captures the temperature dependence of the perylene dye, resulting from the nonradiative processes of its interactions with the surrounding polymer matrix.

**Figure 1:**
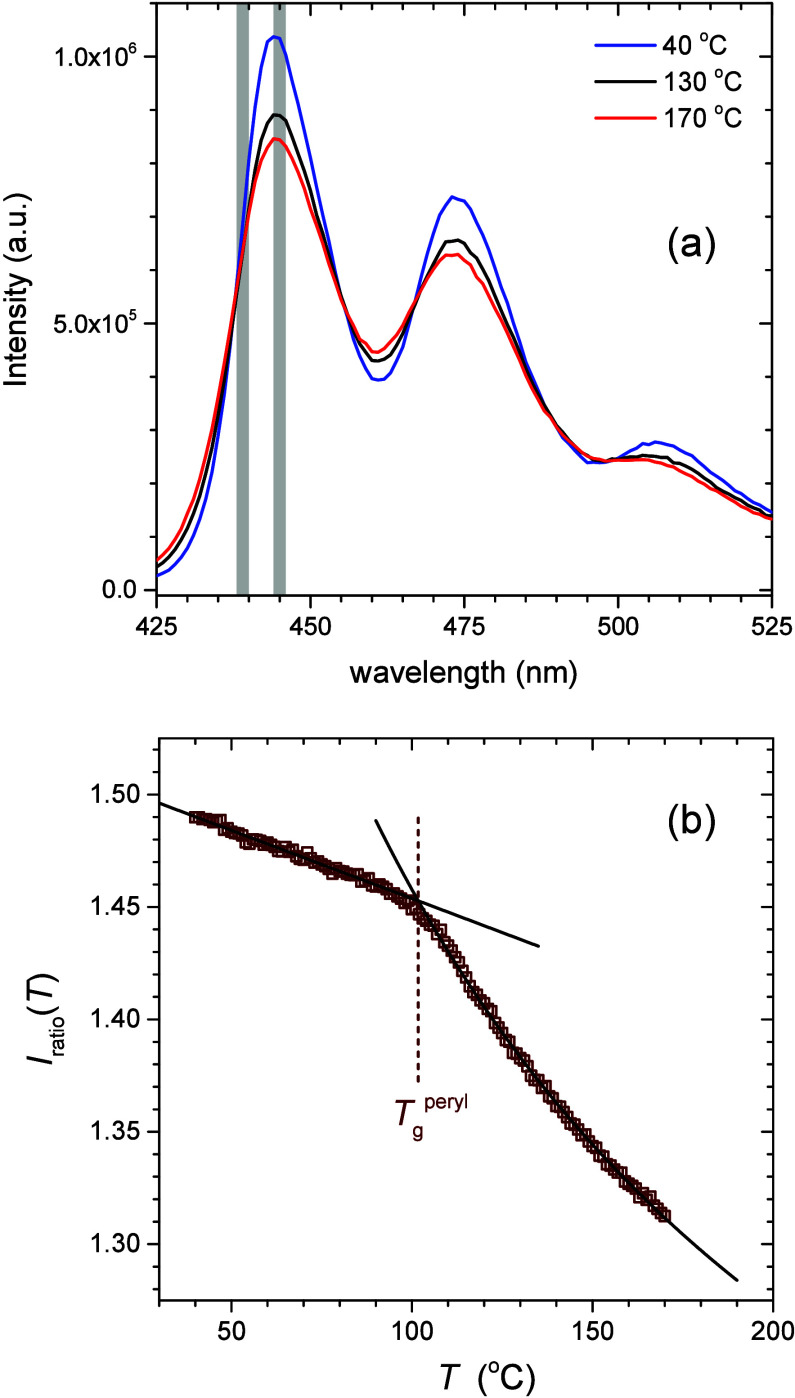
(a) Perylene fluorescence emission spectra collected at 170, 130, and 40 °C for a 1000 nm thick PS film. Gray bars highlight the wavelength regions of the first peak and the temperature-invariant self-referencing region (SRR) to the left of the first peak that are used to calculate the intensity ratio *I*_ratio_ = *I*_peak_/*I*_SRR_. (b) Temperature dependence of *I*_ratio_ for the same 1000 nm thick PS film, where the intersection of fits in the glassy and liquid regime are used to identify the perylene determined glass transition temperature $T_{g}^{\mathrm{peryl}}$ = 102 °C.

In Figure 1b, we plot the temperature dependence of this intensity ratio *I*_ratio_(*T*) collected from the 1000 nm thick PS film. We find that the data show distinctly different temperature dependencies in the glassy and liquid regimes. In the glassy regime, *I*_ratio_(*T*) is linear with temperature, while above *T*_g_, *I*_ratio_(*T*) exhibits a curvature we find is well fit by a WLF functional form. The intersection of two such fit curves occurs at a temperature of 102 ± 2 °C, close to the known *T*_g_ of bulk PS films. We designate this perylene determined glass transition temperature as $T_{g}^{\mathrm{peryl}}$ . We note with our improvements in data collection in the present work we are able to more clearly visualize the change in temperature dependence at *T*_g_ when directly plotting *I*_ratio_(*T*), compared to the previous work of ref 87.

### 3.2 Temperature Dependence of Fluorescence Quantum Yield

The quantum yield of a fluorescent dye

1 $$\Phi =\frac{I_{F}}{I_{E}}$$

corresponds to the fraction of light intensity emitted by fluorescence *I*_F_ relative to the excitation light intensity *I*_E_, reflecting the relative probability of the dye to decay by fluorescence instead of some nonradiative decay pathway. It can also be written as the ratio of the rate of radiative decay to the sum of both radiative and nonradiative decay rates as^63^

2 $$\Phi =\frac{k_{R}}{k_{R}+k_{\mathrm{NR}}}$$

The radiative decay rate *k*_R_ is typically treated as temperature invariant, as it simply reflects electronic energy level transitions.^72,73,91,92^ Thus, the temperature dependence arises primarily from the rate of nonradiative decay *k*_NR_(*T*), reflecting perylene dye’s sensitivity to the molecular vibrations of the surrounding polymer.

The temperature dependence of the nonradiative decay rate can be written as the sum of a temperature independent and temperature dependent contribution as *k*_NR_(*T*) = *k*_0_ + *k*_1_(*T*).^72,73,87^ Previous experimental work has demonstrated that the nonradiative decay rate of dyes in a glassy matrix or in solution is well described by an Arrhenius temperature dependence.^72,73,91,92^ More recent work from our lab has found that the Arrhenius temperature dependence is only observed when the polymer matrix surrounding the dye is below *T*_g_ in the glassy state, while a non-Arrhenius temperature dependence is observed above *T*_g_ when the polymer matrix is in the rubbery state.^87^ As such

3 $$k_{\mathrm{NR}}\left(T\right)=k_{0}+k_{1}\left(T\right)=k_{0}+A\exp\left[-\frac{E\left(T\right)}{k_{B}T}\right]$$

where *E*(*T*) is a temperature-dependent activation energy above the polymer matrix *T*_g_ that becomes temperature-independent below *T*_g_.^87^ *k*_B_ is Boltzmann’s constant and *A* is simply a prefactor. From this, the inverse of the quantum yield can be written as

4 $$\Phi^{-1}=\frac{I_{E}}{I_{F}\left(T\right)}=\frac{k_{R}+k_{0}+A\exp\left[-\frac{E\left(T\right)}{k_{B}T}\right]}{k_{R}}$$

The temperature invariant terms in the above equation can be canceled out by defining a quantity Ω­(*T*) as the difference of the inverse of the quantum yield at the desired temperature relative to its value at absolute zero:^72,87^

5 $$\Omega \left(T\right)=\frac{I_{E}}{I_{F}\left(T\right)}-\frac{I_{E}}{I_{F}\left(0\right)}=\frac{A}{k_{R}}\exp\left[-\frac{E\left(T\right)}{k_{B}T}\right]$$

Equation 5 can be cast into a WLF-type form by constructing a ratio of Ω­(*T*) at a reference temperature *T*_ref_ to the desired temperature *T*. For the left-hand side this gives

6 $$\frac{\Omega \left(T_{\mathrm{ref}}\right)}{\Omega \left(T\right)}=\frac{I_{F}\left(T\right)}{I_{F}\left(T_{\mathrm{ref}}\right)}\left[\frac{I_{F}\left(0\right)-I_{F}\left(T_{\mathrm{ref}}\right)}{I_{F}\left(0\right)-I_{F}\left(T\right)}\right]\approx \frac{I_{F}\left(T\right)}{I_{F}\left(T_{\mathrm{ref}}\right)}$$

where the term in square brackets is approximately one when *T* does not deviate too far from *T*_ref_ relative to absolute zero.^72,87^ The right-hand side of eq 5 becomes

7 $$\frac{\Omega \left(T_{\mathrm{ref}}\right)}{\Omega \left(T\right)}=\exp\left[\frac{E\left(T\right)}{k_{B}T}-\frac{E\left(T_{\mathrm{ref}}\right)}{k_{B}T_{\mathrm{ref}}}\right]$$

Therefore, we arrive at

8 $$\frac{I_{F}\left(T\right)}{I_{F}\left(T_{\mathrm{ref}}\right)}=\exp\left[\frac{E\left(T\right)}{k_{B}T}-\frac{E\left(T_{\mathrm{ref}}\right)}{k_{B}T_{\mathrm{ref}}}\right]$$

showing that the temperature dependence of the fluorescence emission intensity reflects the change in the activation energy of the surrounding polymer matrix with temperature. For our experimental measurements, the fluorescence intensity is captured by *I*_ratio_(*T*) giving us

9 $$\log\left[\frac{I_{\mathrm{ratio}}\left(T\right)}{I_{\mathrm{ratio}}\left(T_{\mathrm{ref}}\right)}\right]=\frac{1}{2.303}\left[\frac{E\left(T\right)}{k_{B}T}-\frac{E\left(T_{\mathrm{ref}}\right)}{k_{B}T_{\mathrm{ref}}}\right]$$

where $\frac{1}{2.303}=\log\left(e\right)$ accounts for the conversion from the natural logarithm to base 10.

### 3.3 Calibrating the Temperature Dependence of Perylene’s Intensity Ratio in Polystyrene

Based on eq 9, we graph in Figure 2 the temperature dependence of the intensity ratio *I*_ratio_(*T*) on an Arrhenius plot as a function of 1000/*T* for two representative PS films: a bulk (500 nm) and a thin (30 nm) film. The overall fluorescence intensity of the thinner film is lower, as the total amount of fluorophore present in the thinner film is obviously less. Both data sets exhibit two distinct temperature regimes: a linear Arrhenius trend below the polymer matrix *T*_g_ with a constant, temperature-independent activation energy and a non-Arrhenius temperature-dependent behavior above the matrix *T*_g_. In the glassy state, a linear fit to the data gives a slope of 0.021 K for the bulk 500 nm film and 0.024 K for the thin 30 nm film. To fit the non-Arrhenius temperature-dependent data above *T*_g_, we use a WLF parametrization of the form:

10 $$\log\left[I_{\mathrm{ratio}}\left(T\right)\right]=\frac{-c_{1}^{*}\left(T-T_{\mathrm{ref}}\right)}{c_{2}+\left(T-T_{\mathrm{ref}}\right)}+b$$

where *T*_ref_ was taken to be $T_{g}^{\mathrm{bulk}}=373K$ , leaving three fit parameters: an offset $b=\log\left[I_{\mathrm{ratio}}\left(T_{\mathrm{ref}}\right)\right]$ , the WLF parameter *c*_2_, and an uncalibrated WLF parameter $c_{1}^{*}$ that depends on how fluorescence intensity correlates with the polymer relaxation time. For the representative data sets shown in Figure 2, the best fit values to the data were found to be $c_{1}^{*}=0.15$ and *c*_2_ = 150 K for the 500 nm thick film and $c_{1}^{*}=0.095$ and *c*_2_ = 128 K for the 30 nm thick film. The values of *b* in both cases agree very well with *I*_ratio_(*T*) at *T*_ref_ as expected. From these two fits we define for each data set the intersection of the Arrhenius linear fit below *T*_g_ with the WLF fit above *T*_g_ as the glass transition temperature of the polymer matrix determined by perylene fluorescence $T_{g}^{\mathrm{peryl}}$ . For the data sets shown in Figure 2, $T_{g}^{\mathrm{peryl}}$ = 373 K for the 500 nm bulk film and $T_{g}^{\mathrm{peryl}}=374K$ for the 30 nm thin film.

**Figure 2:**
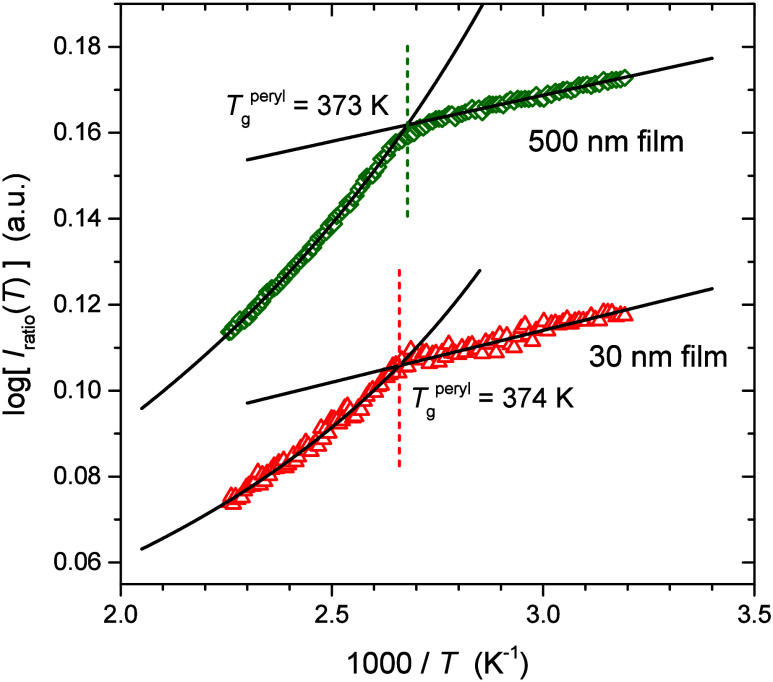
Temperature dependence of *I*_ratio_(*T*) measured from perylene doped in thick and thin PS films. A perylene determined glass transition temperature $T_{g}^{\mathrm{peryl}}$ for the polymer matrix is identified from the intersection of linear fits to the data in the glassy state and WLF fits in the supercooled liquid regime above the polymer’s *T*_g_.

To overlap the data sets across various film thicknesses, we normalize the fluorescence intensity from different films to the value measured at a reference temperature *T*_ref_ taken to be $T_{g}^{\mathrm{bulk}}=373K$ for all films by plotting log­[*I*_ratio_(*T*)/*I*_ratio_(*T*_ref_)]. This factors out overall differences in dye content across samples with varying film thicknesses. Shown in Figure 3 are eight data sets collected from 1000 and 500 nm thick films, demonstrating excellent reproducibility across different samples for bulk films. Bulk fit parameters were identified by performing global fits to these eight data sets simultaneously. The WLF fit above *T*_g_ was to

11 $$\log\left[\frac{I_{\mathrm{ratio}}\left(T\right)}{I_{\mathrm{ratio}}\left(T_{\mathrm{ref}}\right)}\right]=\frac{-c_{1}^{*}\left(T-T_{\mathrm{ref}}\right)}{c_{2}+\left(T-T_{\mathrm{ref}}\right)}$$

giving $c_{1}^{*}=0.16$ and *c*_2_ = 164 K, and the linear fit below *T*_g_ gave a slope of 0.024 K. We can convert the vertical fluorescence intensity axis into meaningful units by correlating the *I*_ratio_(*T*) data to τ­(*T*) data from the literature following

12 $$\log\left[\frac{I_{\mathrm{ratio}}\left(T\right)}{I_{\mathrm{ratio}}\left(T_{\mathrm{ref}}\right)}\right]=c_{f}\log\left[\frac{\tau \left(T\right)}{\tau \left(T_{\mathrm{ref}}\right)}\right]$$

**Figure 3:**
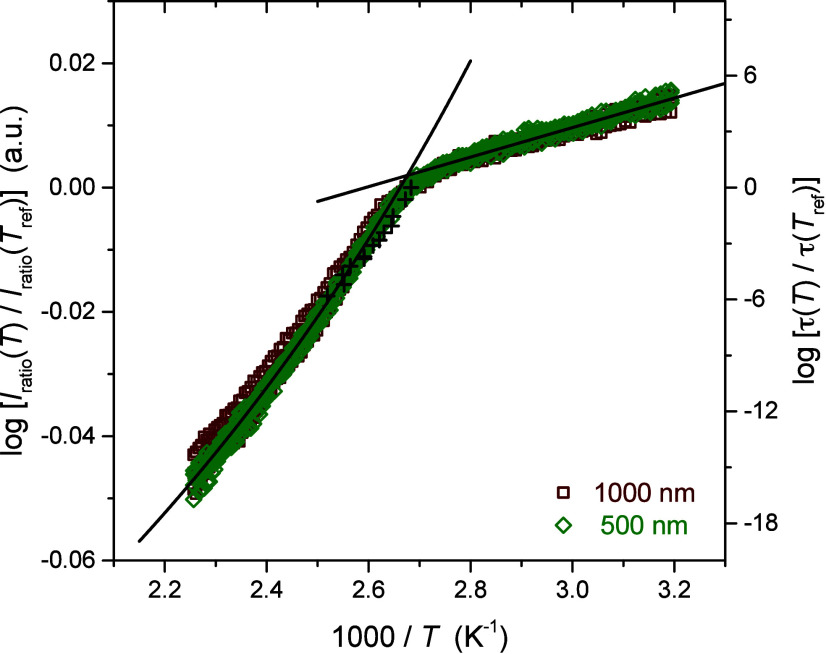
Temperature dependence of the perylene fluorescence intensity *I*_ratio_(*T*) normalized to that at the reference temperature $T_{\mathrm{ref}}=T_{g}^{\mathrm{bulk}}=373K$ for bulk PS films with 500 and 1000 nm across 8 different samples. Global fits to the data were done to obtain bulk fit parameters, which were then correlated to literature τ­(*T*) data by Roland and Casalini^93^ (plotted as black crosses) to arrive at the right vertical axis.

Using dielectric spectroscopy τ­(*T*) data by Roland and Casalini on high molecular weight PS (*M*_w_ = 3840 and 90 kg/mol) over the available temperature range,^93^ we determined the calibration factor *c*_*f*_ = 0.003 from this comparison of bulk films to bulk literature data. Assuming the same correlation of perylene fluorescence intensity to local polymer dynamics holds for all film thicknesses, we calibrate the measured $c_{1}^{*}$ WLF parameter at all film thicknesses using $c_{1}=c_{1}^{*}/c_{f}$ , with *c*_*f*_ = 0.003.

### 3.4 Film Thickness Dependent Behavior of Perylene in Thin Polystyrene Films

We now examine how the temperature dependence of perylene’s fluorescence intensity behavior changes in PS thin films with decreasing film thickness. Plotted in Figure 4 are *I*_ratio_(*T*) data for PS films with thicknesses ranging from 1000 down to 15 nm. The data sets are overlapped by plotting log­[*I*_ratio_(*T*)/*I*_ratio_(*T*_ref_)] versus 1000/*T*, where *T*_ref_ was kept at $T_{g}^{\mathrm{bulk}}=373K$ for all films. Below *T*_g_, the data for all film thicknesses from 15 to 1000 nm overlap well, showing a linear Arrhenius behavior that is independent of the film thickness. Above the polymer matrix *T*_g_, the non-Arrhenius temperature-dependent behavior shows a pronounced trend with decreasing film thickness below ∼500 nm, where notably the 200 nm film already shows deviation from bulk behavior. The thinner films exhibit a remarkably weaker temperature dependence. We use the correlation between *I*_ratio_(*T*) and τ­(*T*) obtained for bulk films using eq 12 to plot log­[τ­(*T*)/τ­(*T*_ref_)] on the right axis of Figure 4. Comparing thick and thin films on this scale, it can be seen that the range of relaxation times spanned with temperature in the liquid state decreases from about 16 orders of magnitude for bulk films to about 8 orders of magnitude for the 15 nm thick films.

**Figure 4:**
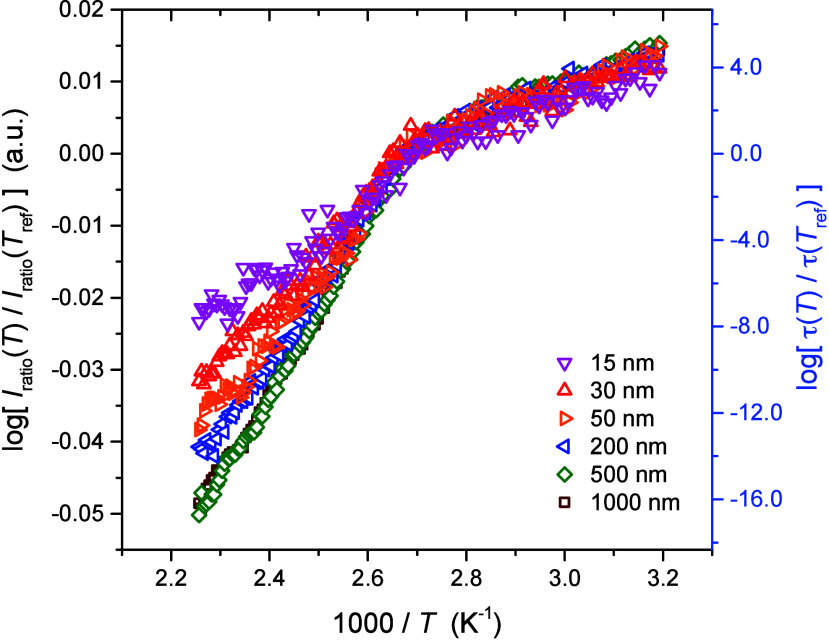
Temperature dependence of the perylene fluorescence intensity *I*_ratio_(*T*) normalized to that at $T_{\mathrm{ref}}=T_{g}^{\mathrm{bulk}}=373K$ for PS films of varying thickness from 15 to 1000 nm, with correlation to the corresponding relaxation time τ­(*T*) on the right axis.

From the data shown in Figure 4, one can also clearly see that the temperature at which the data transition from a non-Arrhenius to an Arrhenius temperature dependence on cooling is the same for all film thicknesses. We fit each fluorescence intensity data set above *T*_g_ to the WLF dependence given in eq 11, resulting in best fit parameters of *c*_2_ and $c_{1}=c_{1}^{*}/c_{f}$ using *c*_*f*_ = 0.003. The fluorescence intensity data below *T*_g_ are fit to an Arrhenius temperature dependence, resulting in a best fit glassy activation energy from the slope in the data of log  *I*_ratio_(*T*) versus 1/*T*:

13 $$E_{G}=\ln10\frac{R}{c_{f}}\left(\mathrm{slope}\right)$$

where *R* is the gas constant. From the intersection of these fits, the perylene determined glass transition temperature $T_{g}^{\mathrm{peryl}}$ is obtained and found to be independent of film thickness for all films measured in the present study, 15 to 1000 nm, where the mean value of $T_{g}^{\mathrm{peryl}}$ = 373 ± 2 K. In addition, we can also calculate the fragility *m* from *T*_g_ = $T_{g}^{\mathrm{peryl}}$ and the WLF parameters we evaluated at *T*_ref_ = $T_{g}^{\mathrm{bulk}}$ using^94,95^

14 $$m{\equiv \frac{d\log\tau }{d\left(T_{g}/T\right)}|}_{T=T_{g}}=\left(\frac{c_{1}}{c_{2}}\right)T_{g}^{\mathrm{peryl}}$$

Figure 5 plots these various fit parameters as a function of the film thickness. As expected from the overlap in the log­[*I*_ratio_(*T*)] data shown in Figure 4, $T_{g}^{\mathrm{peryl}}$ is independent of film thickness to within experimental error. Given the correlation between *I*_ratio_(*T*) and τ­(*T*) from eq 12, it appears that perylene provides a dynamic measure of *T*_g_ comparable to how τ­(*T*) behaves with decreasing temperature. This suggests that the film thickness independence of $T_{g}^{\mathrm{peryl}}$ is an indication that the dynamic glass transition $T_{g}^{\mathrm{dyn}}$ is also independent of film thickness in thin films, consistent with other dynamic measures of *T*_g_ in confined systems.^7,37–39^ We can also see from the data in Figure 4 that the glassy activation energy *E*_G_ obtained from the slope of the data below *T*_g_ is independent of film thickness with an average value of 141 ± 16 kJ/mol. The value we measure for the bulk films of *E*_G_ = 150 ± 12 kJ/mol is in excellent agreement with the activation energy of 146–167 kJ/mol reported for the β-relaxation of bulk PS believed to represent motions of the phenyl groups.^96,97^

**Figure 5:**
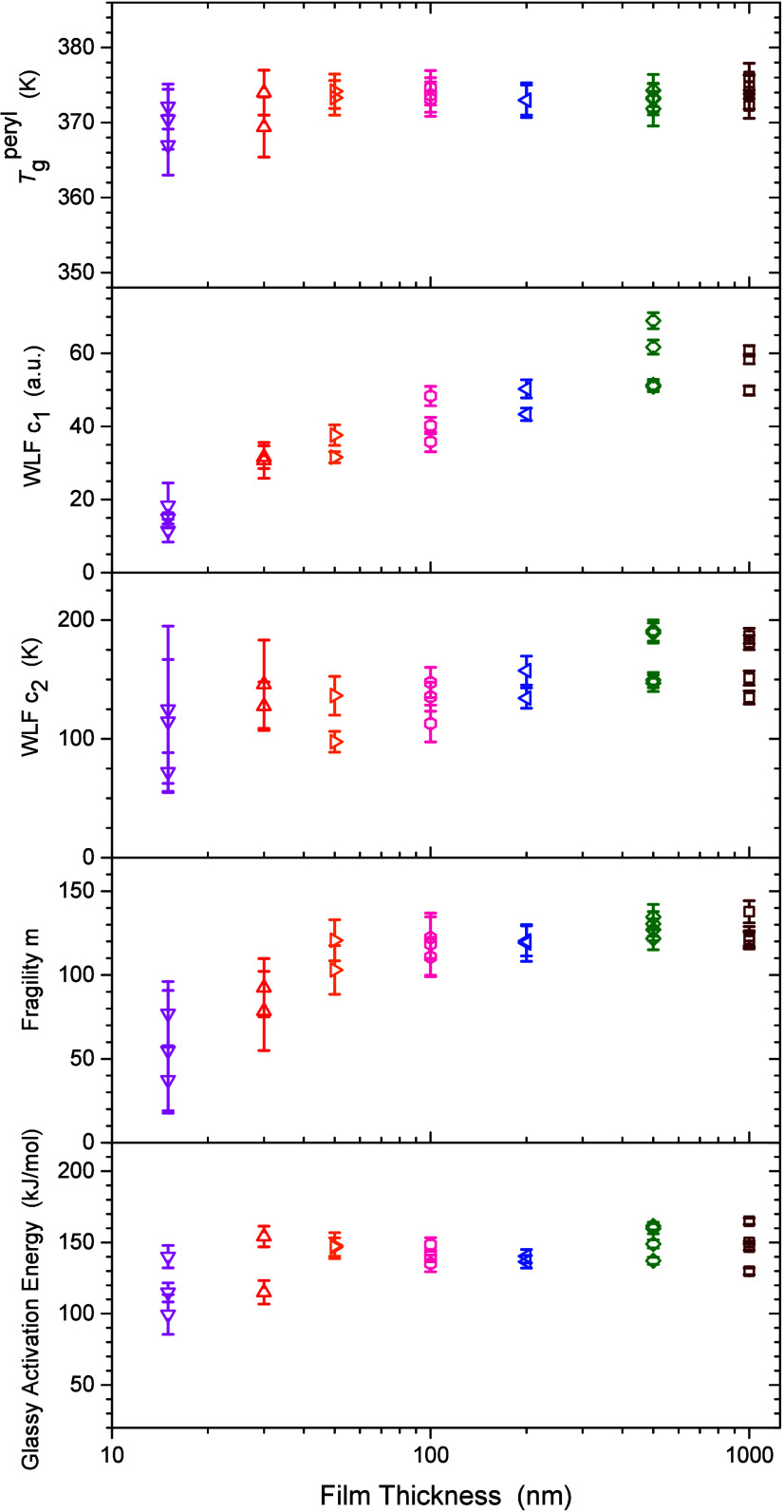
Film thickness dependence of various fit parameters: $T_{g}^{\mathrm{peryl}}$ , WLF parameters *c*_1_ and *c*_2_, fragility *m*, and glassy activation energy *E*_G_.

From the WLF fits to the data above *T*_g_, we find that the parameter *c*_2_ is on average independent of the film thickness. This makes sense given that *c*_2_ reflects the horizontal temperature position of the data (as plotted for instance in Figure 2 and 4). When comparing the WLF and Vogel–Fulcher–Tammann (VFT) equations, *c*_2_ at *T*_g_ corresponds to the difference between *T*_g_ and the Vogel temperature *T*_VFT_, *c*_2_ = *T*_g_ – *T*_VFT_.^95^ In contrast, the WLF parameter *c*_1_ is found to decrease with decreasing film thickness, reflecting the change in the curvature of the relaxation behavior in the liquid state observed in Figure 4. From eq 14, we see that the change in *c*_1_ directly reflects the change in fragility *m*, as $T_{g}^{\mathrm{peryl}}$ and *c*_2_ are not changing with decreasing film thickness. Fragility represents the steepness of the temperature dependent slope of the relaxation dynamics τ­(*T*) as the glass transition *T*_g_ is approached from above.^98^ As we can clearly see from the data in Figure 4, the slope of the data is weakened in thin films, resulting in the decrease in fragility *m* observed in Figure 5. We find that the fragility decreases from 127 ± 6 for the bulk PS films of 500 and 1000 nm, down to 56 ± 20 for the 15 nm thick films. Similar reductions in fragility with decreasing film thickness have been previously reported for thin PS films,^8,10,99^ as well as in molecular dynamics simulations.^11,12^

The WLF parameter *c*_1_ corresponds to the range of relaxation times between τ_α_ at *T*_g_ and the microscopic relaxation time:^100^ *c*_1_ = log­(τ_α_/τ_0_). As the microscopic relaxation time τ_0_ reflects the inherent local time scale of the system, we would expect this to be independent of film thickness. This then suggests that τ_α_ = τ­(*T*) at *T*_g_ decreases with decreasing film thickness, consistent with the dynamics being faster in thin films. To visualize this more clearly, we replot the data of Figure 4 by overlapping the *I*_ratio_(*T*) data at the highest temperature measured 170 °C, as shown in Figure 6. It has been suggested from both experiments and simulations that at high temperature the relaxation dynamics of thin and bulk films merge,^34,41^ as such it seems reasonable to assume that the relaxation dynamics are independent of film thickness at high temperature. Normalizing the *I*_ratio_(*T*) data at 170 °C for the PS films of different thicknesses by plotting log­[*I*_ratio_(*T*)/*I*_ratio_(*T* = 443 K)], we are able to make a correspondence to relaxation times log­[τ­(*T*)/τ­(*T* = 443 K)] that allows us to estimate the average τ­(*T*) at $T_{\mathrm{ref}}=T_{g}^{\mathrm{bulk}}=100\mathrm{{}^{\circ}C}$ for the PS films with decreasing film thickness starting from τ­(*T*_g_) = 100 s for bulk films. Figure 6(b) graphs these values showing that the average τ­(*T*_g_) for the thin films decreases with decreasing film thickness relative to τ_bulk_(*T*_g_) = 100 s for bulk films, by over 8 orders of magnitude for the thinnest 15 nm films. This strong decrease in the average relaxation times τ­(*T*) for the thin films is a distinctly different result than the $T_{g}^{\mathrm{dyn}}$ temperature at which the τ­(*T*) temperature dependence changes from non-Arrhenius to Arrhenius on cooling that is independent of film thickness.

**Figure 6:**
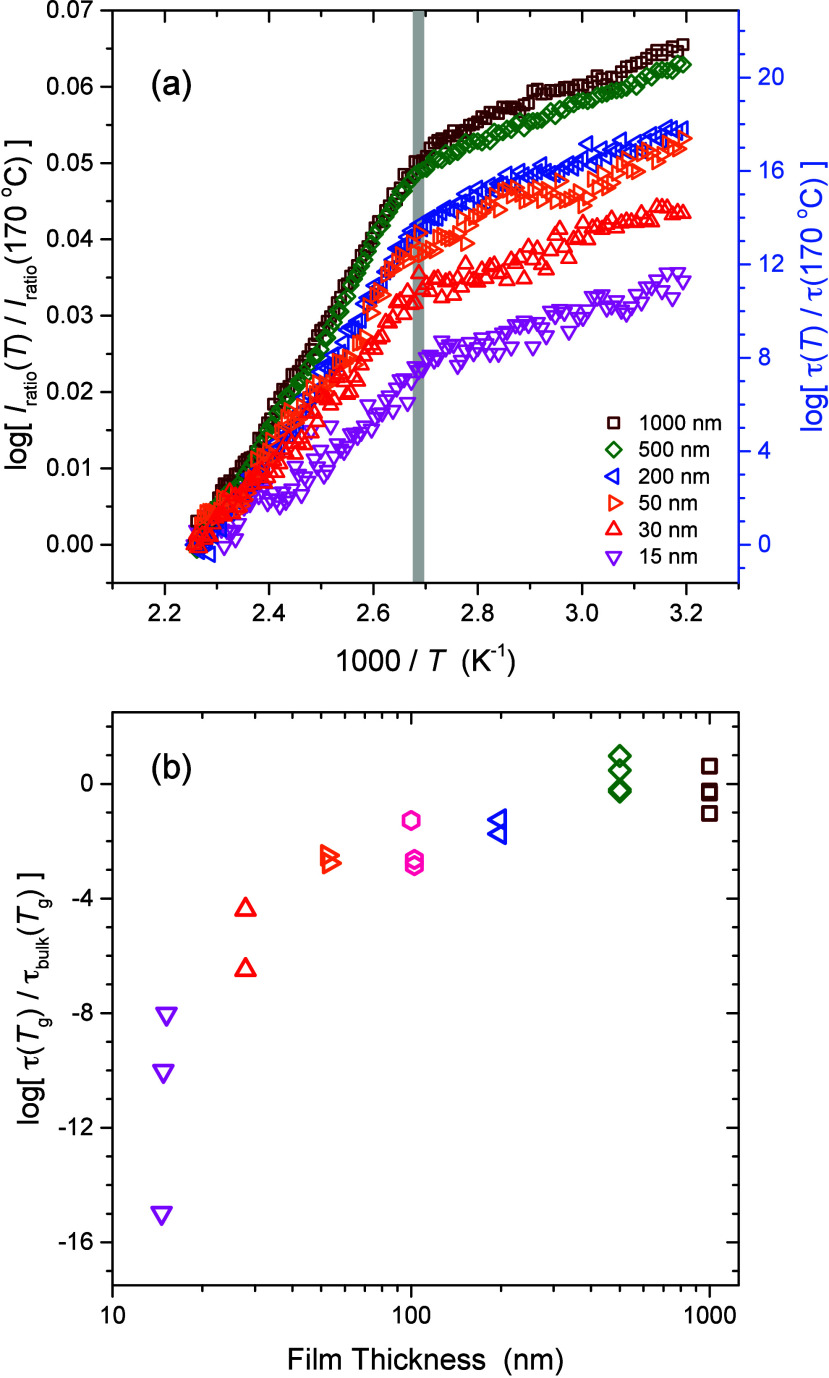
(a) Temperature dependence of perylene fluorescence intensity log­[*I*_ratio_(*T*)/*I*_ratio_(*T* = 443 K)] for PS films of varying thicknesses from 15 to 1000 nm, where the data have been normalized at the highest temperature of 170 °C. Right *y*-axis shows the corresponding relaxation times log­[τ­(*T*)/τ­(*T* = 443 K)]. (b) Film average values of log­[τ­(*T*_g_)/τ_bulk_(*T*_g_)] for the PS films with decreasing thickness.

### 3.5 Contrasting the Response of Perylene and Pyrene Dyes

In Figure 7, we directly compare the film thickness dependence of the glass transition temperatures $T_{g}^{\mathrm{peryl}}$ as measured by perylene and $T_{g}^{\mathrm{pyr}}$ , as measured by pyrene in PS films with decreasing film thickness. We plot the film thickness dependent $T_{g}^{\mathrm{pyr}}$ data collected from our lab in ref 88 using pyrene-labeled PS with *M*_w_ = 582 kg/mol, *M*_w_/*M*_n_ = 1.58, and 0.34 mol % label-content. We also include the two $T_{g}^{\mathrm{pyr}}$ measurements for bulk 500 nm thick films from this study using pyrene doped in PS. The values of $T_{g}^{\mathrm{pyr}}$ were determined from the intersection of linear fits to the fluorescence intensity data above and below *T*_g_ collected on cooling at 1 °C/min. The dashed curve is the best fit equation provided by Keddie, Jones, and Cory to their *T*_g_(*h*) data measured by ellipsometry for PS thin films:

15 $$T_{g}\left(h\right)=T_{g}^{\mathrm{bulk}}\left[1-{\left(\frac{A}{h}\right)}^{\delta }\right]$$

with *A* = 3.2 nm and δ = 1.8.^5^ The solid curve corresponds to the best fit values of *A* = 3.2 nm and δ = 1.63 provided by Ellison et al. to their thin PS film data measured by doped pyrene.^42^ Pyrene dye has long since been shown to exhibit a decrease in *T*_g_(*h*) with decreasing film thickness consistent with the original ellipsometry measurements by Keddie, Jones and Cory,^31,42^ as shown in the figure. In contrast, we observe with perylene dye that the measured glass transition temperature $T_{g}^{\mathrm{peryl}}$ is by comparison independent of film thickness in thin PS films. However, we emphasize that for bulk films, perylene reports a value for the glass transition temperature $T_{g}^{\mathrm{peryl}}=373\pm 2K$ in excellent agreement with $T_{g}^{\mathrm{bulk}}$ determined by pyrene fluorescence $T_{g}^{\mathrm{pyr}}=373\pm 1K.$ Thus, the differences between $T_{g}^{\mathrm{peryl}}$ and $T_{g}^{\mathrm{pyr}}$ arise only in thin confined films. We believe these differences in *T*_g_(*h*) trends between perylene and pyrene fluorescence in thin films reflect how thermodynamic and dynamic measures of *T*_g_ exhibit different behavior in nanoconfined systems, as have been previously reported in the literature.^7,37–40^ Pyrene fluorescence measurements have been considered thermodynamic like, as they agree well with volumetric measures such as ellipsometry, as shown in Figure 7. Whereas, the fluorescence measurements using perylene dye appear to agree well with the trends in dynamic measures of the glass transition $T_{g}^{\mathrm{dyn}}$ observed in refs 8, 37, and 38 that do not exhibit changes in *T*_g_ with confinement.

**Figure 7:**
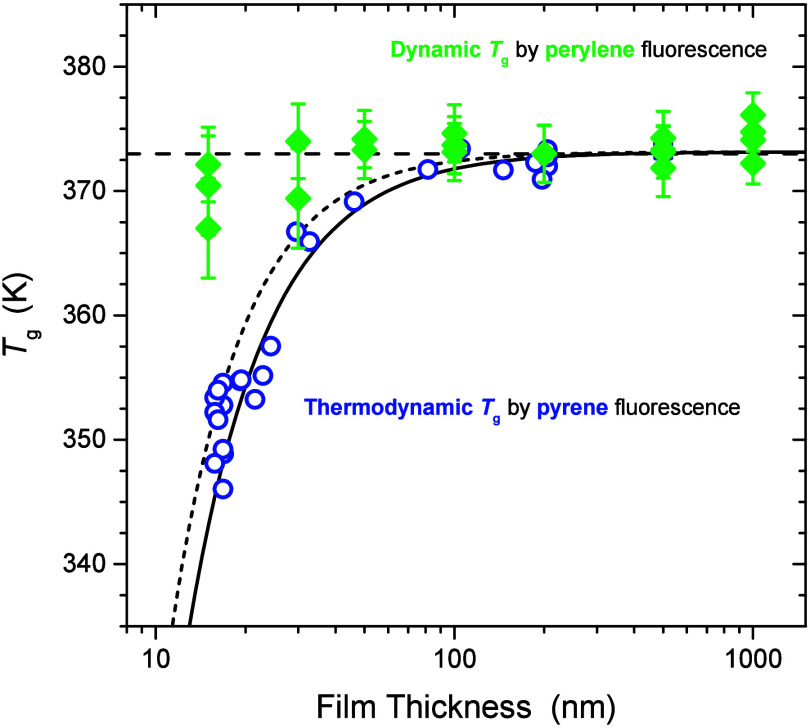
Comparison of the film thickness dependence of the glass transition temperature for PS thin films as measured using two different fluorescent probes: $T_{g}^{\mathrm{peryl}}$ by perylene (green diamonds) and $T_{g}^{\mathrm{pyr}}$ by pyrene from ref 88 (blue circles) and this study (blue squares). Curves correspond to the *T*_g_(*h*) functional form by Keddie, Jones, and Cory (eq 15) using the best fit values from pyrene fluorescence by Ellison et al.^42^ (solid curve) and from ellipsometry by Keddie et al.^5^ (dashed curve). Horizontal dashed line at $T_{g}^{\mathrm{bulk}}=373K$ illustrates how $T_{g}^{\mathrm{peryl}}$ is independent of film thickness.

Differences in the fluorescence temperature dependence between the two dyes reflect how they are each sensitive to their molecular surroundings. Fluorescence intensity corresponds to the fraction of light emitted compared to the loss of energy from the dye’s excited state due to various nonradiative decay processes.^63^ At higher temperatures, more thermal vibrations lead to a higher probability of nonradiative energy loss as the dye is more likely to interact with the molecular vibrations and density fluctuations of its surroundings during its excited state lifetime. The major difference between perylene and pyrene is that perylene has a very short excited state lifetime of 6 ns in comparison to pyrene’s anomalously long lifetime of 450 ns.^63^ Pyrene is known to exhibit sensitivity to the local polarity of its environment because its lifetime is long enough to sample dipole reorientations.^63,101,102^ In contrast, perylene’s short excited state lifetime means it will only be sensitive to high frequency molecular vibrations of order 0.2 GHz or higher. Such high frequency vibrations correspond to elastic fluctuations such as the mean-squared displacement ⟨*u* ^2^⟩ (Debye–Waller factor) of the surrounding polymer material.^103,104^ For example, incoherent neutron scattering that results from inelastic scattering events with random vibrational fluctuations of the material also reflects such high frequencies, typically of order 0.2 GHz and above.^28–30^ Many glass transition theories have correlated the temperature dependence of the relaxation time τ­(*T*) behavior to elastic fluctuations and the Debye–Waller factor ⟨*u* ^2^⟩ as it diverges above *T*_g_ when cage breaking events occur.^105–107^ These elastic models ascribe the activated hopping event of the α relaxation to temporary density fluctuations on the time scale of GHz that allow for a momentary local cage expansion facilitating the local cooperative rearrangement. Perylene’s nonradiative decay rate would be sensitive to these high frequency elastic vibrations that are effectively the precursors of α relaxations.

In addition, elastic fluctuations have also been correlated with penetrant diffusion and ionic conductivity, as a “dynamic free volume” formulation.^108–110^ The relevant activation energy needed for this activated diffusion process depends on the penetrant size.^108,109^ The glassy state activation energy *E*_G_ ≈ 150 kJ/mol we reported in Figure 5 would correspond to the diffusion of relatively small penetrants the size of the styrene monomer itself and not the larger perylene dye,^109^ supporting the assessment that perylene fluorescence is reflecting the motions of the surrounding PS segments. For comparison, our previous work investigating the temperature dependent fluorescence characteristics of perylene doped into different polymers found the value of $T_{g}^{\mathrm{peryl}}$ to vary with the polymer matrix *T*_g_ from 99 °C in P2VP to 152 °C in PC.^87^ These factors strongly support the conclusion that perylene fluorescence captures the vibrational density fluctuations of the surrounding polymer matrix, providing an indirect measure of the dynamic free volume in polymers.

## 4 Conclusions

We demonstrated how perylene fluorescence spectroscopy provides a sensitive and robust probe of polymer dynamics in supported PS thin films. Perylene’s short 6 ns fluorescence lifetime results in the dye’s sensitivity to molecular density fluctuations of the surrounding polymer at GHz frequencies. The temperature dependence of the fluorescence intensity captures changes in the nonradiative energy loss of the excited perylene dye resulting from polymer dynamics within its excited state lifetime. We quantified perylene’s fluorescence using an intensity ratio *I*_ratio_ = *I*_peak_/*I*_SRR_ between the first peak and a temperature-independent self-referencing region (SRR) that can account for changes associated with photobleaching or instrument variability. We showed how the *I*_ratio_(*T*) reflects the temperature-dependent activation energy of the local polymer matrix.

Above the polymer’s glass transition, *I*_ratio_(*T*) follows a non-Arrhenius temperature dependence that was well fit by a WLF functional form. By comparing *I*_ratio_(*T*) for bulk PS films to dielectric relaxation data of bulk PS from the literature, we were able to correlate *I*_ratio_(*T*) with τ­(*T*) in order to convert fluorescence intensity counts to meaningful units. The *I*_ratio_(*T*) data above *T*_g_ exhibit a weakening of the temperature dependence in thin PS films with decreasing film thickness ≤ 200 nm. From the WLF fits, a decrease in the WLF fit parameter *c*_1_ is observed, which is quantified as a decrease in the polymer’s fragility *m* with decreasing film thickness, in agreement with other experimental techniques.^8,10^ Assuming that the local relaxation times at high temperature are independent of film thickness, we are able to quantify the relaxation times τ­(*T*) at *T*_g_ for the different film thicknesses. Relative to bulk, we find that τ­(*T*_g_) decreases by at least 8 orders of magnitude in the thinnest 15 nm films. The Arrhenius temperature dependence of *I*_ratio_(*T*) measured in the polymer’s glassy state results in a glassy state activation energy *E*_G_ = 150 kJ/mol for bulk films that agrees well with reported values for the β-relaxation in PS.^96,97^

The perylene determined glass transition temperature $T_{g}^{\mathrm{peryl}}$ , we defined from the intersection of the WLF fit in the liquid regime and the Arrhenius fit in the glassy regime, is found to be independent of PS film thickness down to 15 nm. This is consistent with other experimental measures of a dynamic glass transition $T_{g}^{\mathrm{dyn}}$ that exhibit no change with increasing nanoconfinement.^8,37–39^ Perylene dye’s $T_{g}^{\mathrm{peryl}}$ response is contrasted with pyrene-determined glass transition values $T_{g}^{\mathrm{pyr}}$ that follow *T*_g_(*h*) decreases in thin PS films in agreement with thermodynamic measures of *T*_g_ such as ellipsometry.^5,31,42,88^ We discussed how this difference in the fluorescence characteristics of the two dyes can be attributed to the large difference in the excited state lifetimes between the two dyes. The contrasting response between perylene and pyrene set the stage for future localized measurements of $T_{g}^{\mathrm{peryl}}$ using a covalently attached perylene dye that should be able to provide significant insight into local differences between dynamic and thermodynamic measures of *T*_g_ in nanoconfined systems.
